# Real‐world drivers of treatment choices in synchronous metastatic renal cell carcinoma

**DOI:** 10.1002/bco2.70149

**Published:** 2025-12-29

**Authors:** Tarik Almdalal, Maja Fahlén, Ulrika Harmenberg, Börje Ljungberg, Magnus Lindskog

**Affiliations:** ^1^ Department of Immunology, Genetics and Pathology Uppsala University Uppsala Sweden; ^2^ Department of Surgery and Urology Eskilstuna County Hospital Eskilstuna Sweden; ^3^ Regional Cancer Centre Stockholm Sweden; ^4^ Department of Pelvic Cancer, Genitourinary Oncology, Karolinska University Hospital, and Department of Oncology‐Pathology Karolinska Institute Stockholm Sweden; ^5^ Department of Diagnostics and Intervention, Urology and Andrology Umeå University Umeå Sweden

**Keywords:** checkpoint inhibitors, cytoreductive nephrectomy, metastatic renal cell carcinoma, surgery, systemic therapy

## Abstract

**Objectives:**

This study aimed to identify clinical and socioeconomic factors associated with treatment selection and survival in patients diagnosed with synchronous metastatic renal cell carcinoma (mRCC).

**Patients and Methods:**

The Renal Cell Cancer Database Sweden (RCCBaSe2.0), linking the National Swedish Kidney Cancer Register with other national quality registers, was used to identify all patients with synchronous mRCC diagnosed 1 January 2014–1 July 2019 (*n* = 951); thus, it was performed during the tyrosine kinase inhibitor era. Logistic and Cox regression were used to evaluate associations with treatment selection, overall survival (OS) and cancer‐specific survival (CSS).

**Results:**

Upfront cytoreductive nephrectomy (uCN) was the primary treatment in 56% of patients and was associated with larger primaries and treatment at university hospitals. Immediate systemic treatment (IST) was chosen in 32% and associated with multidisciplinary team (MDT) discussions, cN1 disease, more metastatic sites and higher comorbidity index. Gender, income, education level or marital status were not associated with upfront treatment. Patients selected for uCN had longer OS and CSS compared with those allocated to IST. This association remained when adjusting for selection factors. Socioeconomic factors were not linked to survival. Limitations include the retrospective design and the lack of detailed data on the International mRCC Database Consortium risk factors.

**Conclusion:**

Tumour‐related factors had significant effects on the choice to perform uCN or not. Patients with more advanced disease, higher comorbidity index and those discussed at MDT were more likely to be offered immediate systemic treatment. Socioeconomic status did not affect treatment allocation or survival, indicating equal healthcare access for Swedish mRCC patients.

## 1 INTRODUCTION

The treatment of patients with synchronous metastatic renal cell carcinoma (mRCC) remains a challenge due to clinical and biological heterogeneity. Surgical resection by upfront cytoreductive nephrectomy (CN) will be potentially curative only if the primary tumour and all metastases are removed. The advantage of CN in the treatment of patients with mRCC was previously shown in a combined analysis including two randomized clinical trials (RCT) comparing CN plus interferon‐based immunotherapy versus interferon only.^1^ When targeted agents, such as tyrosine kinase inhibitors (TKIs) and mTOR inhibitors were introduced, the role of CN has been questioned.

Since 2018, international guidelines have recommended immediate systemic treatment (IST) for most patients with synchronous mRCC according to the International mRCC Database Consortium (IMDC) risk criteria.^2^, ^3^, ^4^, ^5^ The recommendation is based on findings from two contemporary RCTs exploring the treatment with CN and TKI in mRCC. However, both studies were closed in advance, before reaching the intention to treat population of the studies.^2^, ^6^ Their results were supported by a systemic review including an additional 10 non‐RCTs, concluding that IST is prioritized for most patients with mRCC.^7^ Upfront CN has remained an option in patients with a limited disease burden manageable with surveillance or metastasectomy but could also be considered for patients in need of symptom palliation, for example, due to pain or bleeding. Beyond the debate on whether CN should be performed, the optimal timing for CN has also been a matter of discussion.^7^ In clinical practice, each patient needs an individualized treatment decision based on a composite of multiple factors. Given its complexity, the decision‐making could likely be aided by a comprehensive model like this register‐based report to be able to predict the best treatment recommendation.^8^

Real‐world cohorts offer important insights into clinical practice, including decision‐making, treatment feasibility and outcome. Compared to patients treated within clinical trials, real‐world patients are typically older, more comorbid and sociodemographically more heterogeneous.^9^ While studies based on nationwide tumour registries alone may provide population‐based data on disease‐related aspects of treatment selection and survival, they are often hampered by a lack of detail on patient‐related factors likely to affect treatment allocation.

The Swedish database RCCBaSe2.0, which links the National Swedish Kidney Cancer Register (NSKCR) with numerous other national population‐based quality registers, offers unique opportunities to assess the complexity of real‐world mRCC patients. Here, we aimed to identify patient‐, tumour‐, treatment‐related factors associated with treatment selection in a population‐based nationwide cohort of patients with synchronous mRCC and to describe overall survival (OS) and cancer‐specific survival (CSS) in association with treatment allocation.

## 2 PATIENTS AND METHODS

We used the Renal Cell Cancer Database Sweden (RCCBaSe2.0) in which the NSKCR, containing data on 99% of all Swedish patients diagnosed with RCC, was linked with several other national population‐based quality registers: The Swedish Renal Registry, the Swedish Cancer Register, the Prescribed Drug Register, the National Patient Register, the Cause of Death Register, the Longitudinal integrated database for health insurance and labour market studies, the Register of the Total Population, the National Diabetes Register and the Swedish Perioperative Register. The register has near‐complete national coverage (99%), and its validity has been confirmed in a recent study, with most variables showing >90% agreement and very few missing values.^10^

### 2.1 Study population

Patients diagnosed with synchronous mRCC between 1 January 2014 and 1 July 2019 were identified from RCCBaSe2.0, with extraction of patient‐, socioeconomic‐, tumour‐ and treatment‐related factors. Patient‐related variables included age, sex, disposable income, education level, marital status and Charlson comorbidity index (CCI) unadjusted for age.^11^ Notably, CCI was calculated from information obtained from the well‐validated Swedish national patient registry and hence did not rely on reporting by the treating medical units. Tumour‐related variables included tumour size, clinical T‐stage, clinical N‐stage, number of organs with metastatic involvement and tumour histology. Treatment‐related factors included discussion at a multidisciplinary team (MDT) conference and type of hospital (university hospital vs county hospital).

The type of primary treatment was defined as upfront cytoreductive nephrectomy (uCN), IST or best supportive care (BSC). The RCCBaSe2.0 database did not contain granular data on the composition of immediate oncological treatments. The study period was limited to the TKI monotherapy era prior to the approval of immunotherapy‐based combinations in Sweden. CSS and OS were defined as the time from diagnosis to death from RCC or any cause, respectively, or to last follow‐up.

### 2.2 Statistical analysis

Associations of pretreatment factors with choice of primary treatment were analysed using logistic regression and strengths of associations were presented as odds ratios (OR) with 95% confidence intervals (CI). Cox regression was used to construct uni‐ and multivariate survival models defining hazard ratios (HR) for death due to RCC or any cause with 95% CI. We applied the Benjamini–Hochberg procedure to control the false discovery rate (FDR) across all predictors in the four regression models.^12^ Adjusted *p*‐values are reported, with *p* ≤ 0.05 considered statistically significant. Survival data were graphically presented as Kaplan–Meier curves. Individual disposable annual income was inflation‐adjusted and expressed relative to the national median income, categorized into three groups: low (<60% of the median), middle (60%–120%), and high (>120%). CCI was derived using information about diagnoses up to 7 years prior to the RCC diagnosis. The number of organs with metastatic involvement was assessed using data within 3 months prior to, and up to the RCC diagnosis. All analyses were performed using R Statistical Software (v4.3.0; R Core Team 2023).

### 2.3 Ethical approval

The study was approved by the regional Ethical Review Board of northern Sweden EPM: Dnr 2012–418‐31 M; Dnr 2019–02579; Dnr 2020–05093; Dnr 2023–04365‐02.

## 3 RESULTS

A total of 951 patients diagnosed with synchronous mRCC were identified. Table 1 shows patients' baseline characteristics. Tumour morphology was predominantly clear cell RCC (73%), and most tumours were clinical stage T3 (39%). Clinical lymph node metastases (cN1), diagnosed by computed tomography, were present in 326 patients (34%). Upfront CN was the primary treatment in 528 (56%) patients, IST in 304 (32%), and BSC in 119 (13%).

**TABLE 1:** Patient‐, tumour‐ and treatment‐related baseline characteristics among 951 patients with synchronous metastatic renal cell carcinoma shown in relation to the primary treatment offered between 1 January 2 014 and 1 July 2 019.

Variable		uCN	IST	BSC	Total
Patients	No	528	304	119	951
Age (years)	Mean (range)	67 (32–86)	69 (39–89)	77 (35–94)	69 (32–94)
Gender	Males	349 (56%)	195 (31%)	77 (12%)	621 (100%)
	Female	179 (54%)	109 (33%)	42 (13%)	330 (100%)
Married	Yes	287 (56%)	173 (34%)	51 (10%)	511 (100%)
	No	236 (54%)	130 (30%)	68 (16%)	434 (100%)
	Unknown	5	1	0	6
Higher education	Yes	122 (66%)	49 (27%)	13 (7%)	184 (100%)
	No	395 (53%)	250 (33%)	103 (14%)	748 (100%)
	Unknown	11	5	3	19
Income	Low	70 (53%)	46 (35%)	15 (11%)	131
	Medium	219 (50%)	150 (33%)	84 (19%)	453
	High	234 (65%)	107 (29%)	20 (6%)	361
	Unknown	5	1	0	6
Tumour size (mm)	Mean ± CI	92 ± 4	80 ± 4	89 ± 16	88 ± 3
	Unknown	0	8	9	17
Clinical T‐Stage	TX	3	59	20	82
	T1	82	93	30	205
	T2	74	93	35	202
	T3	314	38	18	370
	T4	55	21	16	92
Clinical N‐Stage	NX	39	36	21	96
	N0	344	140	45	529
	N1	145	128	53	326
No of metastatic sites	Mean ± CI	1.3 ± 0.2	1.6 ± 0.1	1.5 ± 0.2	1.4 ± 0.1
RCC‐type	ccRCC	447	196	52	695
	chRCC	10	1	2	13
	pRCC	36	15	5	56
	Other	33	53	17	103
	Unknown	2	39	43	84
CCI*	Mean ± CI	1.6 ± 0.2	2.7 ± 0.4	3.3 ± 0.6	2.2 ± 0.2
University hospital	Yes	239	90	32	361
	No	289	214	87	590
MDT	Yes	410	285	73	768
	No	117	18	44	179
	Unknown	1	1	2	4

*Note*: Participants with a registered partnership were categorized as married, while participants with a dissolved registered partnership were categorized unmarried.

Abbreviations: BSC, best supportive care; CCI, Charlson comorbidity index unadjusted for age; ccRCC, clear cell RCC; chRCC, chromophobe RCC; IST, immediate systemic therapy; MDT, multidisciplinary treatment conference; pRCC, papillary RCC; RCC, renal cell carcinoma; uCN, upfront cytoreductive nephrectomy; unknown, missing values.

Compared with patients offered CN or IST, those with BSC were generally older, had more comorbidities, were more likely to have cN1 disease and were less frequently discussed at MDT (Table 2). Larger primary tumours and treatment at a university hospital favoured uCN. In contrast higher comorbidity index, cN1 disease, higher number of metastatic sites and MDT favoured IST, while age, gender, marital status, educational level or income were not associated with the choice of treatment in the adjusted analysis.

**TABLE 2:** Factors associated with choice of treatment for 951 patients with synchronous metastatic renal cell carcinoma.

	Variable	Univariate, unadjusted			Multivariate, adjusted			
		OR	95% CI	*p*‐value	OR	95% CI	*p*‐value	*p*‐adj*
a **uCN or IST vs BSC**	Age (years)	0.88	0.85–0.91	***p* < 0.001**	0.88	0.85–0.91	***p* < 0.001**	**p = 0.005**
	Gender: Male	1.01	0.66–1.54	*p* = 0.962	0.58	0.34–0.96	***p* = 0.038**	*p* = 0.079
	Married	1.44	0.96–2.17	*p* = 0.079	1.32	0.82–2.13	*p* = 0.247	*p* = 0.359
	Higher education	2.10	1.17–4.12	***p* = 0.020**	1.43	0.74–2.98	*p* = 0.311	*p* = 0.440
	Income							
	‐Medium	Ref			Ref			
	‐High	3.66	2.21–6.36	***p* < 0.001**	1.78	0.97–3.37	*p* = 0.068	*p* = 0.128
	‐Low	1.73	0.95–3.37	*p* = 0.088	1.69	0.82–3.71	*p* = 0.169	*p* = 0.252
	CCI*	0.87	0.81–0.92	***p* < 0.001**	0.87	0.81–0.94	***p* < 0.001**	***p* = 0.005**
	Tumour size (cm)	1.00	0.96–1.05	*p* = 0.879	0.98	0.93–1.03	*p* = 0.455	*p* = 0.562
	Clinical N‐Stage	0.56	0.37–0.85	***p* = 0.006**	0.37	0.22–0.61	***p* < 0.001**	***p* = 0.005**
	No. metastatic sites	0.86	0.68–1.13	*p* = 0.259	0.87	0.64–1.18	*p* = 0.349	*p* = 0.482
	University hospital	1.52	0.99–2.38	*p* = 0.061	1.47	0.90–2.45	*p* = 0.135	*p* = 0.212
	MDT	3.21	2.06–4.94	***p* < 0.001**	3.62	2.13–6.15	***p* < 0.001**	***p* = 0.005**
b **uCN vs IST**	Age (years)	0.98	0.97–1.00	***p* = 0.013**	0.98	0.96–1.00	*p* = 0.072	*p* = 0.131
	Gender: Male	1.06	0.78–1.44	*p* = 0.693	1.15	0.81–1.63	*p* = 0.446	*p* = 0.562
	Married	0.96	0.72–1.28	*p* = 0.778	1.16	0.83–1.62	*p* = 0.380	*p* = 0.501
	Higher education	1.57	1.08–2.29	***p* = 0.019**	1.58	1.03–2.43	***p* = 0.037**	*p* = 0.079
	Income							
	‐Medium	Ref			Ref			
	‐High	1.46	1.07–2.00	***p* = 0.018**	1.18	0.80–1.74	*p* = 0.397	*p* = 0.512
	‐Low	1.06	0.68–1.66	*p* = 0.799	1.01	0.60–1.69	*p* = 0.983	*p* = 0.983
	CCI*	0.88	0.83–0.92	***p* < 0.001**	0.89	0.84–0.94	***p* < 0.001**	***p* = 0.005**
	Tumour size (cm)	1.10	1.05–1.14	***p* < 0.001**	1.12	1.07–1.18	***p* < 0.001**	***p* = 0.005**
	Clinical N‐Stage	0.52	0.38–0.70	***p* < 0.001**	0.55	0.39–0.78	***p* = 0.001**	***p* = 0.005**
	No. metastatic sites	0.52	0.42–0.63	***p* < 0.001**	0.48	0.37–0.60	***p* < 0.001**	***p* = 0.005**
	University hospital	1.97	1.45–2.69	***p* < 0.001**	1.74	1.24–2.46	***p* = 0.001**	***p* = 0.005**
	MDT	0.17	0.09–0.29	***p* < 0.001**	0.16	0.08–0.30	***p* < 0.001**	***p* = 0.005**

*Note*: Results from unadjusted and adjusted logistic regression models comparing (a) upfront cytoreductive nephrectomy and immediate systemic therapy, with best supportive care and (b) upfront cytoreductive nephrectomy with immediate systemic therapy. *p‐adj, *p*‐values adjusted using Benjamini–Hochberg procedure to control the false discovery rate across all predictors. Due to missing data (a) 46, respectively, (b) 33 observations were excluded. Significant *p*‐values are given in **bold**.

Abbreviations: BSC, best supportive care; CCI, Charlson comorbidity index unadjusted for age; IST, immediate systemic therapy; MDT, multidisciplinary team conference; MDT, multidisciplinary team conference; uCN, upfront cytoreductive nephrectomy.

Among the 528 patients planned for upfront CN, 12 (2%) ultimately did not undergo surgery. None of the CN patients died within 30 days postoperatively, while 26 (5%) died within 90 days. In patients receiving IST, five of 304 patients (2%), died within 30 days and 38 (13%) died within 90 days. Notably, 16 (5%) of the 304 patients with IST later underwent deferred CN, with a median time to surgery of 13 months.

### 3.1 Cancer‐specific and OS

**FIGURE 1:**
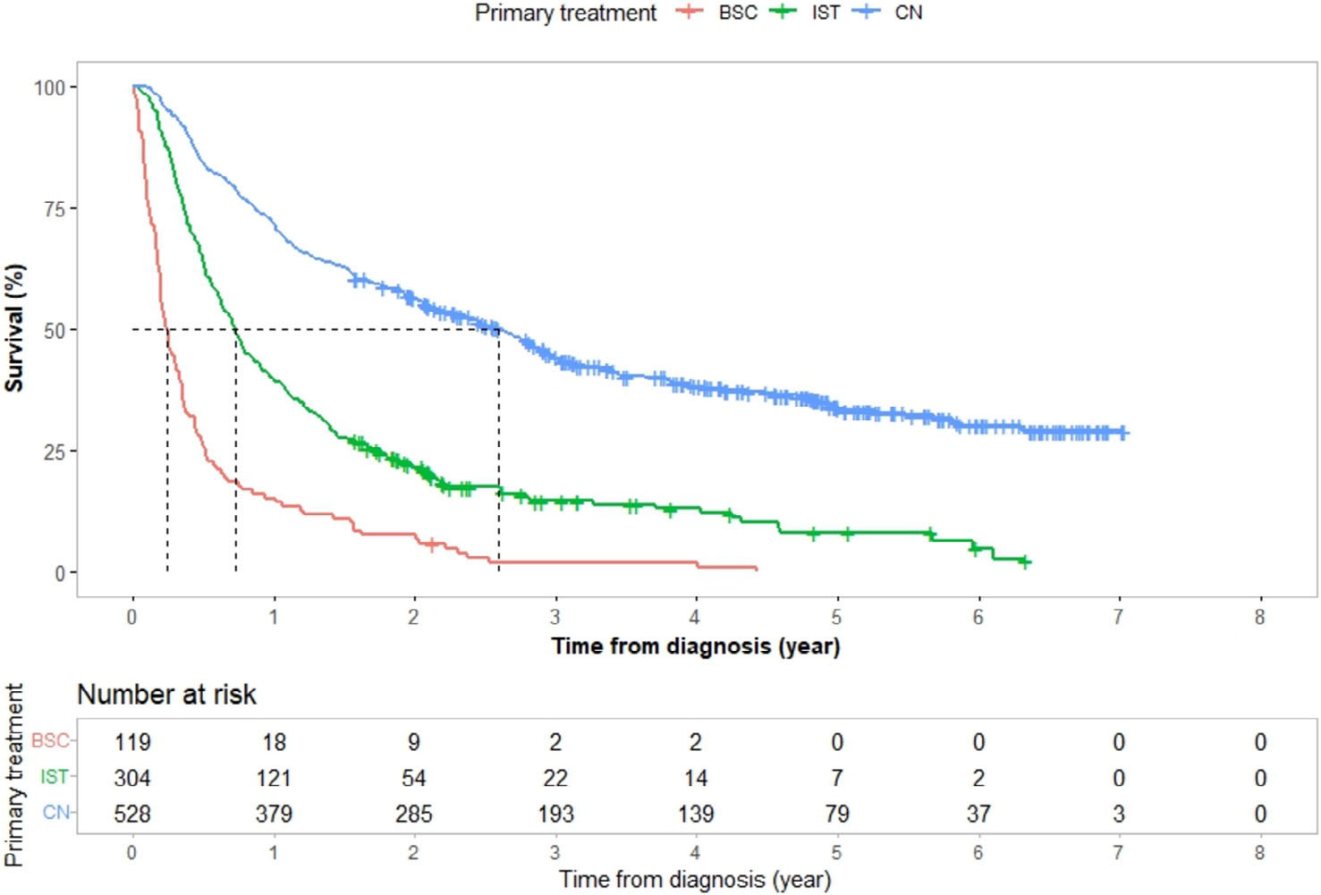
Kaplan–Meier curves showing the overall survival for 951 patients with synchronous metastatic renal cell carcinoma stratified by primary treatments: upfront cytoreductive nephrectomy (CN, blue), immediate systemic treatment (IST, green) and best supportive care (BSC, red). The x‐axis shows time from diagnosis in years, and the y‐axis shows the proportion of surviving patients. Overall survival was significantly longer in the CN group compared with IST and BSC. Dashed lines indicate median survival times. The table below the graph shows the number of patients at risk at each time point.

**FIGURE 2:**
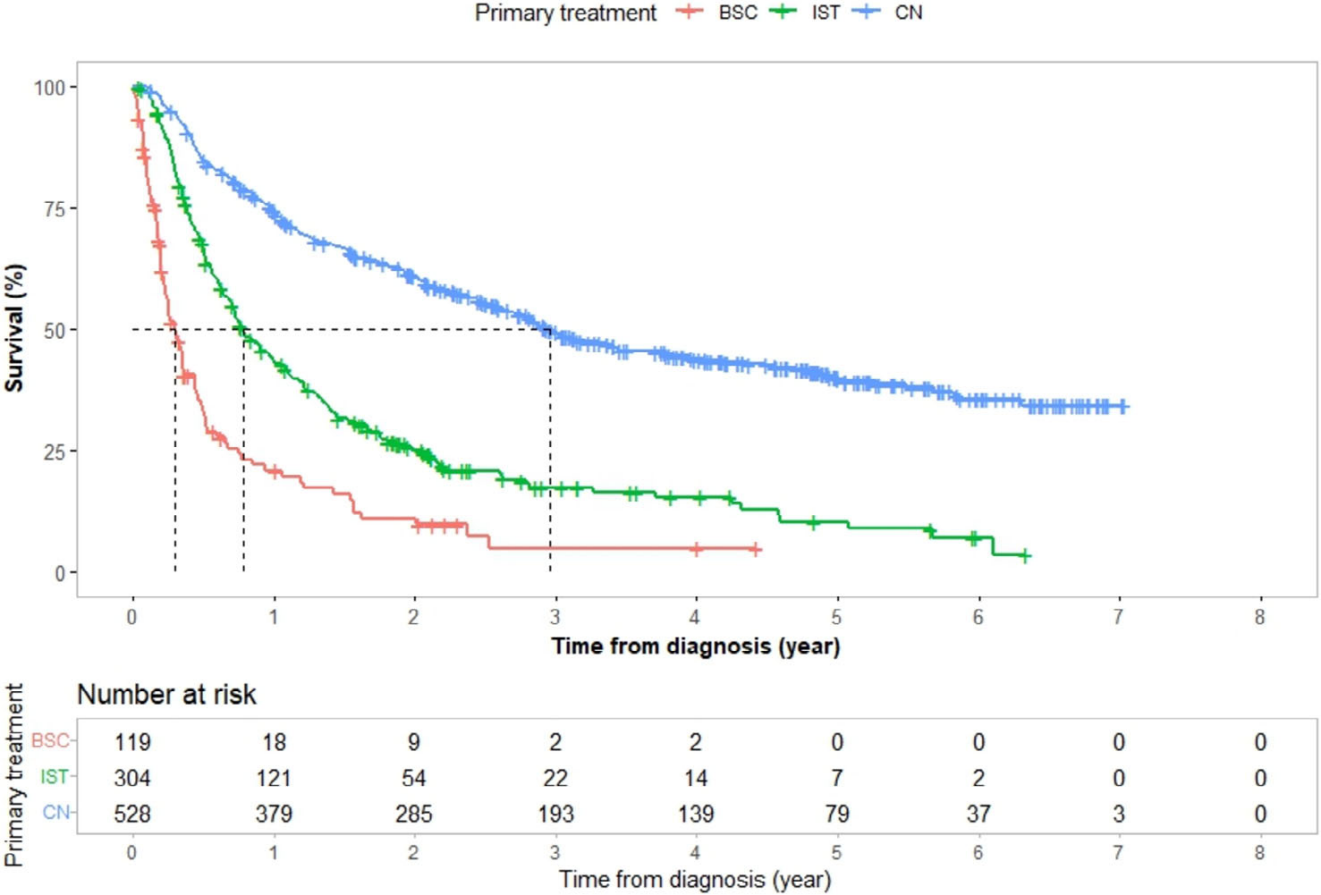
Kaplan–Meier curves showing cancer‐specific survival for 951 patients with synchronous metastatic renal cell carcinoma stratified by primary treatment: upfront cytoreductive nephrectomy (CN, blue), immediate systemic treatment (IST, green) and best supportive care (BSC, red). The x‐axis shows time from diagnosis in years, and the y‐axis shows the proportion of surviving patients without RCC mortality. Cancer‐specific survival was significantly longer in the CN group compared with IST and BSC. Dashed lines indicate median survival times. The table below the graph shows the number of patients at risk at each time point.

Patients selected for uCN had a longer median survival than those receiving IST, median OS 31 versus 9 months (95% CI 25–36 vs. 7–11) and median CSS 35 versus 9 months (95% CI 31–46 vs. 8–12), respectively, as shown in Figures 1 and 2. The treatment choice remained linked to survival—even after controlling for factors associated with treatment allocation. Results from Cox regression models for OS and CSS are presented in Table 3. In multivariate adjusted analysis, CCI, tumour size, cN‐stage, number of metastatic sites, other RCC‐types and BSC, all associated independently to worse OS and CSS times. Patients selected for uCN, and patients with chromophobe RCCs had significantly better survival, while age, gender, marital status, income, educational level, hospital type or MDT had no independent association to survival (Table 3). Patients selected to uCN had about 50% lower risk of death compared with patients selected to IST, with adjusted HRs of 0.51 (95% CI 0.42–0.63) for OS and 0.50 (95% CI 0.40–0.62) for CSS.

**TABLE 3:** Results from the adjusted and unadjusted cox regression models for time from primary diagnosis of 828 patients with metastatic renal cell carcinoma (RCC) to overall death and to RCC‐specific death, respectively.

Event	Variable	Univariate, unadjusted			Multivariate, adjusted			
		HR	95% CI	*p*‐value	HR	95% CI	*p*‐value	*p*‐adj *
**Overall death**	Age (years)	1.01	1.00–1.02	*p* **= 0.005**	1.01	1.00–1.02	*p* = 0.090	*p* = 0.154
	Gender: Male	0.94	0.80–1.12	*p* = 0.499	0.87	0.73–1.04	*p* = 0.139	*p* = 0.213
	Married	0.92	0.79–1.08	*p* = 0.330	0.88	0.74–1.03	*p* = 0.120	*p* = 0.194
	Higher education	0.73	0.59–0.90	***p* = 0.003**	0.82	0.66–1.02	*p* = 0.078	*p* = 0.138
	Income							
	‐ Medium	Ref			Ref			
	‐ Low	0.97	0.76–1.25	*p* = 0.833	0.96	0.74–1.26	*p* = 0.792	*p* = 0.851
	‐ High	0.83	0.70–0.99	***p* = 0.034**	1.07	0.87–1.30	*p* = 0.530	*p* = 0.615
	CCI*	1.10	1.07–1.14	***p* < 0.001**	1.09	1.06–1.12	***p* < 0.001**	***p* = 0.005**
	Tumour size (cm)	1.02	1.00–1.04	*p* = 0.082	1.04	1.02–1.06	***p* < 0.001**	***p* = 0.005**
	Clinical N‐Stage	1.97	1.67–2.33	***p* < 0.001**	1.66	1.38–1.99	***p* < 0.001**	***p* = 0.005**
	No metastatic sites	1.52	1.38–1.67	***p* < 0.001**	1.28	1.15–1.42	***p* < 0.001**	***p* = 0.005**
	RCC‐type ‐ ccRCC	Ref			Ref			
	‐ chRCC	0.31	0.12–0.83	***p* = 0.020**	0.26	0.09–0.74	***p* = 0.012**	***p* = 0.028**
	‐ pRCC	1.13	0.82–1.58	*p* = 0.454	1.16	0.83–1.63	*p* = 0.378	*p* = 0.501
	‐ Other RCC‐types	2.37	1.88–2.99	***p* < 0.001**	1.53	1.20–1.95	***p* = 0.001**	***p* = 0.005**
	University hospital	0.83	0.70–0.98	***p* = 0.024**	1.00	0.84–1.18	*p* = 0.977	*p* = 0.983
	MDT (yes or no)	1.15	0.93–1.42	*p* = 0.188	1.01	0.81–1.27	*p* = 0.905	*p* = 0.955
	Primary treatment							
	‐ IST	Ref			Ref			
	‐ uCN	0.40	0.33–0.47	***p* < 0.001**	0.51	0.42–0.63	***p* < 0.001**	***p* = 0.005**
	‐ BSC	2.28	1.73–3.00	***p* < 0.001**	2.55	1.88–3.45	***p* < 0.001**	***p* = 0.005**
**RCC**‐**specific death**	Age (years)	1.01	1.00–1.01	*p* = 0.230	1.00	0.99–1.01	*p* = 0.636	*p* = 0.710
	Gender: Male	0.90	0.76–1.08	*p* = 0.268	0.83	0.68–1.00	*p* = 0.051	*p* = 0.102
	Married	0.90	0.76–1.07	*p* = 0.224	0.87	0.73–1.04	*p* = 0.117	*p* = 0.194
	Higher Education	0.72	0.57–0.90	***p* = 0.003**	0.80	0.63–1.01	*p* = 0.060	*p* = 0.116
	Income							
	‐ Medim	Ref			Ref			
	‐ Low	1.01	0.77–1.33	*p* = 0.933	0.95	0.71–1.27	*p* = 0.725	*p* = 0.794
	‐ High	0.88	0.73–1.05	*p* = 0.162	1.08	0.87–1.33	*p* = 0.483	*p* = 0.572
	CCI*	1.08	1.05–1.12	***p* < 0.001**	1.07	1.04–1.11	***p* < 0.001**	***p* = 0.005**
	Tumour size (cm)	1.03	1.01–1.05	***p* = 0.004**	1.04	1.03–1.06	***p* < 0.001**	***p* = 0.005**
	Clinical N‐Stage	2.07	1.73–2.47	***p* < 0.001**	1.71	1.41–2.08	***p* < 0.001**	***p* = 0.005**
	No metastatic sites	1.54	1.39–1.71	***p* < 0.001**	1.26	1.12–1.41	***p* < 0.001**	***p* = 0.005**
	RCC‐type ‐ ccRCC	Ref			Ref			
	‐ chRCC	0.36	0.13–0.97	***p* = 0.043**	0.28	0.09–0.82	***p* = 0.021**	***p* = 0.047**
	‐ pRCC	1.13	0.79–1.61	*p* = 0.503	1.14	0.79–1.64	*p* = 0.482	*p* = 0.572
	‐ Other RCC‐types	2.27	1.77–2.92	***p* < 0.001**	1.49	1.15–1.94	***p* = 0.003**	***p* = 0.008**
	University hospital	0.79	0.66–0.95	***p* = 0.011**	0.95	0.79–1.14	*p* = 0.592	*p* = 0.674
	MDT (yes or no)	1.18	0.94–1.49	*p* = 0.149	1.01	0.79–1.29	*p* = 0.935	*p* = 0.969
	Primary treatment							
	‐ IST	Ref			Ref			
	‐ uCN	0.39	0.33–0.48	***p* < 0.001**	0.50	0.40–0.62	***p* < 0.001**	***p* = 0.005**
	‐ BSC	1.98	1.46–2.69	***p* < 0.001**	2.35	1.68–3.28	***p* < 0.001**	***p* = 0.005**

*Note*: Hazard ratio (HR) and 95% confidence interval (CI) for overall survival and RCC‐specific survival are shown, analysing age at diagnosis, sex, comorbidity, RCC‐type, university hospital, multidisciplinary team conference and primary treatment method. 123 observations were excluded due to missing data. * *p*‐values are adjusted using Benjamini–Hochberg procedure to control the false discovery rate (FDR) across all predictors in the regression models. Significant *p*‐values are given in **bold**.

Abbreviations: BSC, best supportive care; CCI, Charlson Comorbidity index, unadjusted for age; ccRCC, clear cell RCC; chRCC, chromophobe RCC; CI, confidence intervals; HR, hazard ratio; IST, immediate systemic treatment; MDT, multidisciplinary team conference; pRCC, papillary RCC; RCC, renal cell carcinoma; uCN, upfront cytoreductive nephrectomy.

## 4 DISCUSSION

We present a nationwide, population‐based study of patients with synchronous mRCC in Sweden 2014–2019. The most important novel contribution of this study to the field, compared with previous register studies,^13^, ^14^, ^15^ is the use of the RCCBaSe2.0, which enabled us to control for potentially confounding, patient‐related factors, commonly not available in most conventional tumour registries. Our primary objective was to understand what clinical factors govern selection of uCN versus IST in real‐world patients with synchronous mRCC. We found that lymph node involvement and more metatstatic sites favoured IST. Larger primary tumours and treatment at a university hospital and favoured uCN. The association between treatment at a university hospital and uCN may reflect referral bias of patients with more locoregionally advanced disease and less metastatic burden. In contrast, patients with more advanced comorbidity were more likely to receive IST, in line with a lower likelihood of being good surgical candidates.

It is reasonable to expect MDT discussions to refine treatment selection in synchronous metastatic cancers. Our finding of an association between MDT and IST might indicate that MDTs make teams less likely to send mRCC patients straight to upfront CN but more likely to favour IST in accordance with guidelines recommendations.^4^ Despite affecting allocation to primary therapy, we found no evidence of an independent prognostic role of MDT discussions in patients with synchronous mRCC, despite refinement and individualization of treatment selection for each patient. In contrast, in a Chinese study, MDT was associated with prolonged OS in a smaller single center study including both synchronous and metachronous mRCC patients.^16^ This discrepancy could potentially be influenced by different triggers for MDT in the Chinese and Swedish settings.

Studies addressing the role of socioeconomic factors in mRCC are relatively rare. Here, we investigated whether educational level, income or marital status affected upfront treatment allocation in synchronous mRCC. We found no such associations. In contrast, a register study from the United States, using the National Inpatient Sample database between 2006 and 2021, found reduced CN utilization in synchronous mRCC patients with lower income.^17^ Furthermore, other US studies found associations between race, income, insurance coverage, hospital type and marital status with offered treatments.^18^, ^19^ Another recent population‐based study by Metcalf et al. found that socioeconomic disparities not only affect treatment decision‐making for CN but also differences in survival rates for different population groups.^20^ Notably, the tax‐funded Nordic health care systems are intended to be universal and guided by the principle of ‘care on equal terms’. Nevertheless, people with lower socioeconomic status have been shown to experience worse cancer‐related outcomes than higher groups in the Nordics as well.^21^ In the present study, neither educational level, income nor marital status were associated with OS or CSS. We consider that these findings indicate equal healthcare access nationwide for Swedish mRCC patients.

We found that patients selected for uCN showed prolonged OS and CSS compared with IST. At least in part, this probably reflects selection bias of patients considered likely to benefit from uCN and for whom systemic therapy could be safely postponed. Interestingly, the longer survival associated with uCN remained when controlling for age, comorbidity, tumour size and N‐stage. As expected, patients receiving BSC had substantially worse outcomes than either active treatment group.

In the RCCBaSe2.0, we limited our inclusion period until to June 2019, which coincides with the approval date for first line immunotherapy in Sweden. Consequently, in real‐life practice, all patients in our cohort who received IST can be expected to have received a single‐agent TKI in first line. The oncological treatment in our cohort study thereby aligns well with the systemic treatments given in the CARMENA and the SURTIME studies.^2^, ^6^ Awaiting prospective RCTs with immunotherapy backbone both these trials remain the basis for the EAU guidelines recommendation, to offer IST to intermediate‐ and high‐risk patients.^4^ However, the current treatment recommendations for mRCC patients, is immunotherapy‐based therapies, either immunotherapy doublets or TKI/immunotherapy regimens.^4^ Notably, a recent register study in clear cell mRCC found that patients treated with CN, regardless of whether they also received immunotherapy, had better survival than those treated with immunotherapy alone.^22^ In the pivotal ICI studies, most patients with mRCC had their primary tumours in place, and post hoc subgroup analyses have provided no clear answer as to whether this affects outcome or not. RCT in this setting, such as the ongoing NORDIC‐SUN trial, aim to address this issue.^23^ Meanwhile, data from the IMDC^24^ and a recent Danish register study^14^ support uCN as a possible treating option in selected patients. Another study reported that patients treated with CN had better results no matter treated with upfront or delayed surgery.^25^ A recent real‐world Dutch study on synchronous mRCC, showed a preference for uCN in patients with mRCC with favourable prognostic factors despite combinations with TKI or immunotherapy.^26^

A drawback of the RCCBaSe2.0 is the lack of detailed information on IMDC risk groups, which were not reported to the NSKCR. By definition, all patients in our cohort were in the IMDC intermediate or high‐risk groups due to synchronous mRCC. The possibility of making a distinction between IMDC intermediate and poor risk patients would have increased the clinical utility of our findings further. However, we were able to control for several aspects of tumour burden, including the number of metastatic sites, as well as a validated comorbidity index.

Our study also provides insights regarding the timing of CN in the targeted therapy era. Only 5% of patients who initially received systemic treatment later underwent deferred CN, suggesting that this option was used only infrequently during the study period. Compared with the SURTIME study,^6^ we observed no mortality during the first 30 postoperative days in CN patients. This was further supported by an Italian study showing a significantly higher 30 days postoperative complication rate in patients undergoing deferred CN compared to upfront CN.^25^ This uncertainty regarding the optimal timing for CN in relation to possible systemic therapy, was also mentioned by Patel et al.^27^

The main strength of this study compared to previous register studies of mRCC is the uniqueness of the RCCBaSe2.0, which enabled us to control patient‐related factors, including comorbidity and socioeconomic factors, data typically lacking in conventional tumour quality registries. Moreover, >99% of all RCC patients in Sweden during the study period were included in the RCCBaSe2.0 permitting excellent coverage of close to all synchronous mRCC cases. Limitations include the lack of detailed information of the systemic TKI therapy used, the absence of a distinction between IMDC intermediate and poor risk and the restriction of study period to the TKI era. Further limitations include the lack of randomization and its register‐based design.

In conclusion, we have evaluated the relevance of several tumour‐ and patient‐related factors on decision‐making to perform uCN or give IST in synchronous mRCC in Sweden. Socioeconomic status was not linked with treatment allocation or survival, indicating equal healthcare access in our health care system. Upfront CN was associated with beneficial OS and CSS. This keeps uCN as a treatment option in selected patients with synchronous mRCC. Randomized prospective trials are awaited to evaluate the role and optimal timing of CN in the immunotherapy era.

## AUTHOR CONTRIBUTIONS

Conception and design: M.L., B.L., T.A. Acquisition of data: M.F., B.L., U.H. Analysis and interpretation of data: M.F., B.L. Drafting of the manuscript: T.A., M.L. Critical revision of the manuscript for important intellectual content: U.H., B.L., M.F., M.L., T.A. Statistical analysis: M.F., B.L., T.A. Obtaining funding: T.A. Administrative support: B.L. Supervision: M.L., U.H., B.L.

## CONFLICT OF INTEREST STATEMENT

Tarik Almdalal, Maja Fahlen and Börje Ljungberg declare no conflict of interest. Ulrika Harmenberg received a research grant from Ipsen. Magnus Lindskog has participated in the advisory board of BMS, Ipsen, MSD, Pfizer and Eisai; received a research grant from Ipsen; and has been invited as lecturer for MSD, Ipsen, Pfizer and BMS.
